# Differences in cardiorespiratory fitness, physical activity, and pulmonary function across occupational groups and educational levels: a cross-sectional study

**DOI:** 10.1080/07853890.2026.2638086

**Published:** 2026-03-05

**Authors:** Natalia Chróścielewska, Tomasz Chomiuk, Katarzyna Laprus-Abramska, Diana Pałasz, Artur Mamcarz, Daniel Śliż

**Affiliations:** a 3rd Department of Internal Medicine and Cardiology, Medical University of Warsaw, Warsaw, Poland; b Centre of Comprehensive Rehabilitation (CKR), Konstancin Jeziorna, Poland

**Keywords:** Blue-collar workers, cardiorespiratory fitness, physical activity, pulmonary function, white-collar workers

## Abstract

**Background:**

Insufficient physical activity (PA) and low cardiorespiratory fitness (CRF) are risk factors for lifestyle diseases, including cardiovascular diseases. Lifestyle plays a major role in shaping CRF and overall health, and professional work is an important part of this.

**Aims:**

To examine associations between the type of work, industry, level of education, weekly working hours, and self-reported PA, CRF, pulmonary function using questionnaire-based assessment, submaximal exercise testing, and spirometry.

**Methods:**

A survey was conducted on occupation, hours worked per week, night shift work, education, age, and gender, as well as a PA survey based on the IPAQ-SF. The Åstrand–Rhyming fitness test was used to estimate *V*O_2max_. Spirometry was performed to assess pulmonary function, with results including FVC, FEV_1_, and the FEV_1_/FVC ratio. The group consisted of 203 professionally active adults aged 40–70 (55.17% men, 39.41% blue-collar workers, mean age: 53.4).

**Results:**

Blue-collar workers reported higher self-reported weekly PA (*p* = 0.0003), and this result was significant for men (*p* = 0.0003). White-collar workers had higher estimated *V*O_2max_ (*p* = 0.0467), but when stratified by gender, only female blue-collar workers had lower values than female white-collar workers (*p* = 0.0142). Among industries the lowest self-reported weekly PA values were observed in male representatives of the professionals group, and the highest in agriculture and forestry workers.

**Conclusions:**

Among men, blue-collar workers reported higher self-reported weekly PA than white-collar workers, with no significant differences in estimated *V*O_2max_. Among women, blue-collar workers did not report higher self-reported PA but had lower estimated *V*O_2max_ than white-collar women. These differences are small and should be interpreted cautiously.

## 1. Introduction

Physical inactivity is defined as engaging in less physical activity (PA) than is necessary to meet the current guidelines for age. Insufficient PA is a significant risk factor for mortality, metabolic syndrome, type II diabetes, mental illness, and, above all, cardiovascular diseases (CVD) [1,2].

According to World Health Organization (WHO) recommendations, adequate PA is at least 150 min of moderate-intensity aerobic exercise or 75 min of vigorous aerobic exercise per week, or a combination of both types of activity. In addition, muscle-strengthening exercises (resistance training or strength training) of moderate or high intensity are recommended for at least 2 days per week. The recommendations also include reducing the amount of time spent sitting in favour of PA, even at low intensity. Greater health benefits can be achieved by being active for at least 300 min per week [3].

Meeting WHO recommendations for PA reduces the risk of CVD by 7% between the ages of 45 and 85 (46% compared to 53%) compared to physically inactive men, and by 11% in women (31% compared to 42%) [4].

Cardiorespiratory fitness (CRF) is an important parameter predicting morbidity and CVD risk. The results of a study by Blaha et al. indicated that higher CRF (measured by MET value) is associated with lower mortality, lower risk of heart attack, and younger biological age. Among the study participants, despite a decline in fitness with age, there was still a strong relationship between this parameter and the incidence of heart attack and mortality [5]. Spirometry is the gold standard test for pulmonary diseases and can provide important markers of CVD, particularly FEV_1_ and FVC parameters [6–8]. Although studies combining spirometry results with *V*O_2max_ are limited, there is data suggesting that pulmonary function influences exercise capacity and a relationship between spirometry parameters and *V*O_2max_ [9,10]. Spirometry testing combined with CRF testing provides a more comprehensive view of cardiovascular function.

CVDs are the most common cause of morbidity and death worldwide. According to statistics, in Poland in 2021, these diseases were responsible for 35% of all deaths [11]. In the same year, CVD accounted for one-third of deaths worldwide [12]. The main risk factors for the development of cardiovascular disease that can be modified include smoking, hypertension, elevated blood glucose levels, lipid disorders, obesity, unhealthy diet, and physical inactivity. Lifestyle is therefore crucial in shaping cardiovascular health and its deterioration [13].

Professional work is an extremely important element of a lifestyle. On average, work occupies about one-third of the day, making it an important lifestyle component with a significant impact on an individual’s health. The health of workers is affected by several work-related factors, including the conditions of the work environment, physical and psychological strain, and prolonged forced body positions. The literature also points to the indirect effect of work activity on health through its association with socio-economic status [14]. Inequalities in the health of the working population have important implications for organizational performance, affecting the health status of human resources, levels of sickness absence, and work performance [15]. According to Eurostat, Poles rank third in Europe in terms of the average number of working hours per week, at 39.3 h. Only the Greeks (39.8 h) and Romanians (39.5 h) have a higher average working time [16].

Blue-collar and white-collar occupations differ in both the level of physical activity and the nature of muscular system involvement. The type of work performed therefore influences body composition, physical fitness levels, and, consequently, overall health, including the risk of cardiovascular disease. Given the serious health, social and economic consequences, education in prevention and epidemiological surveillance of obesity and cardiovascular disease is one of the key challenges of modern medicine [17]. However, blue-collar and white-collar workers encompass many different industries and occupations that vary in terms of PA and skill level [18].

White-collar work is mostly sedentary and is associated with lower levels of PA [19]. White-collar work has been associated with a higher risk of metabolic syndrome, hypertension, and cardiovascular disease in several studies [20–22], which is potentially related to sedentary behavior and occupational stress. In contrast, the term blue-collar work usually refers to occupations categorized as working class, which are characterized by considerable physical and often psychological strain. This type of employment is associated with several adverse health effects, including chronic stress, low job satisfaction, disadvantaged socio-economic position, and increased prevalence of risk factors such as smoking and obesity [18,23]. Taken together, both white- and blue-collar work carry occupational health risks, though the specific risk profiles differ between groups.

Available research indicates the important role of education level in shaping an individual’s overall health. Adults with higher levels of education show better health and live longer compared to their peers with lower levels of education [24]. Higher education is positively correlated with better health indicators, both subjective and objective [25]. Another significant risk factor for work-related CVD is shift work. Night work particularly contributes to the development of inflammation [26]. In addition, a significant increase in blood pressure and a weaker drop in blood pressure are also observed in people working night shifts [27]. This is mainly caused by circadian rhythm disruption, which in turn causes physiological, behavioural, and psychosocial stress, leading to conditions that are risk factors for CVD, such as overweight, obesity, diabetes, and hypertension [28].

This study aimed to examine associations between occupational characteristics (type of work, industry, level of education, and weekly working hours) and PA (self-reported), CRF, and pulmonary function in adults aged 40 years and older, using questionnaire-based assessment, submaximal exercise testing, and spirometry. While numerous studies have examined occupational PA across different job categories, substantially fewer have focused on objective physiological outcomes such as CRF. Moreover, pulmonary function is rarely considered alongside CRF in occupational health research, despite both being integral components of cardiorespiratory function. The combined assessment of these parameters may provide a more comprehensive understanding of health-related adaptations associated with occupational conditions. The present study examines these associations in a cohort of working adults recruited in Poland. Given the morbidity and mortality resulting from physical inactivity and a changing environment, requiring less physical effort, this study may provide valuable information for public health, especially in relation to CVDs. In addition, it may help indicate directions in health prevention programs that can be initiated both at the workplace level and at the national level.

## Materials and methods

A total of 203 individuals staying in the inpatient rehabilitation ward were enrolled in the study. Patient recruitment was carried out personally by the principal investigator.

**Inclusion criteria** included the following: absence of contraindications for exercise testing (according to guidelines by American Heart Association (AHA)) or spirometry (according to guidelines by American Thoracic Society); current employment as a white- or blue-collar worker (with a work interruption not exceeding 6 months at the time of enrollment); return to work following rehabilitation; age between 40 and 70 years.

**Exclusion criteria** included: age below 40 or above 70 years; unemployment or engagement in work other than white- or blue-collar professions; professional inactivity exceeding 6 months at the time of assessment; history of cancer within the previous 5 years; physical or mental disability; presence of chronic diseases in the exacerbation phase; period of less than 6 weeks after orthopedic surgery; and any medical contraindications to the performed tests.

After a preliminary eligibility assessment based on medical records by the principal investigator, participants were interviewed in person to confirm fulfillment of the inclusion criteria. Eligible individuals were subsequently invited to participate in the study. All individuals who met the inclusion criteria agreed to participate.

Participation in the study was voluntary. Written informed consent was obtained from all subjects involved in the study. The study was conducted in accordance with the Declaration of Helsinki and approved by the Bioethics Committee of the Medical University of Warsaw (KB/127/2023).

In terms of occupational type, patient selection was completely random. The study was conducted between November 2023 and February 2025.

The sample size was determined a priori using a power analysis based on the Student’s *t* test for two independent means. Since no published effect size estimates for *V*O_2max_ or pulmonary function were available at the design stage, the calculation drew upon data from the existing literature that reported statistically significant differences in BMI between blue-collar versus white-collar workers [29]. The analyzed cohort is part of a wider study that also assessed body composition, and the same BMI-based sample size was used for the *V*O_2max_ and spirometry analyses presented in this manuscript. Although a minimum of 120 participants per group (blue-collar and white-collar workers) was required to achieve 90% power at an alpha level of 0.05, the final study sample consisted of 203 participants.

Of the 203 qualified individuals, 12 did not complete the cardiorespiratory fitness (CRF) test. The main reasons were: feeling unwell during the test and abnormal blood pressure response to exercise. The missing values of *V*O_2max_ concerned 5 blue-collar workers and 7 white-collar workers. There were 17 missing spirometry results due to repeated incorrect breathing manoeuvres. 6 of the missing data were from blue-collar workers, and 11 were from white-collar workers.

Potential confounders such as body composition and comorbidities were considered in the study design. Participants with significant chronic diseases were excluded according to the inclusion/exclusion criteria, resulting in a relatively healthy, active cohort. Body composition was measured as part of the larger body composition study, but was not included in the analyses presented here to avoid unnecessary complexity. Smoking status was recorded and evaluated as a potential covariate.

The basic questionnaire collected sociodemographic data, including age, gender, occupation, average weekly working hours, education level, and working night shifts. Occupations were classified according to the International Standard Classification of Occupations (ISCO-08) [30]. ISCO-08 uses a four-digit coding system: the first digit denotes the major group (e.g. 5 – services and sales workers), the second represents the sub-major group (e.g. 51 – personal service workers), the third indicates the minor group (e.g. 511 – travel attendants, conductors, and guides), and the fourth specifies the unit group (e.g. 5113 – travel guides).

In this study, only major groups were analyzed: 1 – Managers; 2 – Professionals; 3 – Associate professionals; 4 – Administrative and customer service workers; 5 – Service, care, and sales workers; 6 – Agricultural and forestry workers; 7 – Building and manufacturing workers; 8 – Mechanical manufacturing and transport workers; 9 – Elementary occupations, and 10 – Military personnel.

Since no participants were classified under major group 10, this category was excluded from the analysis. Occupations were grouped into white-collar (major groups 1–5) and blue-collar (major groups 6–9) categories [18].

Educational attainment was categorized according to the Education Law of 14 December 2016 and the Law on Higher Education and Science of 20 July 2018. Based on this classification, education levels included: primary education, lower secondary education, basic vocational education, basic trade education, secondary trade education, secondary education, and higher education. Due to *n* = 0 in the group with lower secondary education and basic vocational education, these groups were excluded from the analysis.

### CRF test

CRF plays an important role in predicting mortality and cardiovascular health implications [5]. CRF, expressed as estimated *V*O_2max_, was assessed using the Åstrand–Rhyming submaximal cycle ergometer test (BTL Ergoselect 5). This test is a reliable tool, cheap and easy to perform, especially when a direct physical performance test is not available. The validity of the Åstrand–Rhyming test for estimating *V*O_2max_ has been well documented in previous studies [31,32].

In this study, heart rate (HR) was monitored continuously and recorded every minute using a chest-worn HR monitor (Polar H9). Before the start of the test, blood pressure was measured using an upper arm blood pressure monitor (BTL ergometer equipment) at rest and monitored during the test and 1 min after the end of the test.

Absolute *V*O_2max_ (L/min) was estimated based on the workload and the average HR. The test lasted six minutes, with participants maintaining a pedaling cadence of 60 ± 5 revolutions per minute (rpm). During the initial two minutes, the workload was adjusted to reach a steady-state HR between 120 and 175 beats per minute, corresponding to approximately 75% of the predicted maximum HR. For each participant, the final workload and acceptable HR were recorded. If HR differed by more than five beats per minute between the fifth and sixth minute, the test was extended until HR stabilized.

Estimated *V*O_2max_ (mL/kg/min) was calculated by converting the absolute *V*O_2max_ to milliliters per minute and dividing it by body weight.

All CRF tests were performed by the same person to ensure methodological repeatability.

### Pulmonary function test

Spirometry assessments were conducted in accordance with the standards set by the American Thoracic Society. Following standard procedure, measurements of forced vital capacity (FVC) and forced expiratory volume in one second (FEV_1_) were obtained from a minimum of three forced expiratory maneuvers [33]. Accordingly, each participant was required to perform three acceptable attempts. Assessments were carried out while participants were seated, using a portable spirometer (EasyOne Air) and a nose clip to ensure accurate readings. Pulmonary function parameters included FVC (% predicted), FEV_1_ (% predicted), and the FEV_1_/FVC ratio (% predicted).

Given the proven negative impact of smoking on spirometry parameters, participants were also asked about the number of cigarettes smoked per day [34,35].

### PA questionnaire

In order to assess the level of PA, a shortened version of the IPAQ-SF questionnaire was used. This questionnaire assesses three main types of PA depending on intensity: walking and moderate, and vigorous intensity. The data are converted into MET-min/week values. Depending on the intensity of the activity, MET (metabolic equivalent) values vary – for walking (3.3 METs), moderate-intensity (4.0 METs), and vigorous-intensity activities (8.0 METs). To calculate the level of PA per week, the following calculation is performed: weekly energy expenditure (MET-min/week) = MET × duration of PA type (min) × frequency [36–38].

### Statistical analysis

All statistical analyses were conducted using SAS software, version 9.4 (SAS Institute Inc., Cary, NC, USA). Statistical significance was set at *p* < 0.05.

Given the overall non-normality of most study variables, all two-group comparisons were performed using the Mann–Whitney *U* test for consistency across analyses. This applies to analyses between groups: blue-collar and white-collar workers, and night shift or no night shift workers.

For the purpose of comparing selected physiological parameters between-group comparisons (industry, education) of FEV_1_, FVC, FEV_1_/FVC, *V*O_2max_ were performed using the non-parametric Kruskal–Wallis test, followed by pairwise post hoc comparisons with the Dwass–Steel–Critchlow–Fligner method.

For variables where significant differences between groups were found in the overall analysis, additional analyses stratified by gender were performed to assess whether the effect differs between women and men.

Quantitative variables are expressed as median and interquartile range (IQR) presented as a single value (Q3–Q1).

To assess the relationship between weekly working hours and PA levels and physiological parameters – FEV_1_, FVC, FEV_1_/FVC, *V*O_2max_ – Spearman’s rank correlation coefficient (ρ) was calculated while controlling for gender.

## Results

Of the 203 participants included in the study, 55.17% were men and 44.83% were women. Blue-collar workers accounted for 39.41% of the total population, while white-collar workers accounted for 60.59%, but there were significantly fewer women among blue-collar workers (12%). The average age of the respondents was 53.4, with men averaging 54.6 and women 51.8. The median age in the white-collar worker group was 53 years, the same as for blue-collar workers. The mean age for blue-collar workers was 53.13, and for white-collar workers 53.59. This is important information, as age is a significant predictor of *V*O_2max_. A correlation was therefore established between these variables and between age and MET-min/week. In the analysis of the entire sample, a moderate, statistically significant correlation was found between age and *V*O_2max_ (*r* = −0.36, *p* < 0.001). Similarly, in the case of MET-min/week, a slight decrease in declared PA was observed with age (*r* = −0.23, *p* < 0.001). However, blue-collar and white-collar workers did not differ in terms of age (χ^2^ = 0.10, *p* = 0.75), which limits the risk of this factor influencing the comparison of *V*O_2max_ and MET-min/week between groups.

Participant numbers for each subgroup are summarized in Tables 1 and 2.

**Table 1.** Demographic characteristics of participants according to type of work (all, white-collar or blue-collar workers).

Variable	Category	All% (*n*)	White-collar% (*n*)	Blue-collar% (*n*)	*p* value
Gender	Men	55.1 (112)	28 (57)	27 (55)	0.0023
	Women	44.8 (91)	32 (66)	12 (25)	
Education	Primary education	2.96 (6)	1.48 (3)	1.48 (3)	<.0001
	Basic vocational education	16.75 (34)	3.45 (7)	13.3 (27)	
	Secondary trade education	12.32 (25)	2.46 (5)	9.85 (20)	
	Secondary education	24.14 (49)	13.79 (28)	10.34 (21)	
	Higher education	43.84 (89)	39.41 (80)	4.43 (9)	
Night shifts	No night shifts	79.3 (161)	50 (102)	29 (59)	0.1555
	Night shifts	20.7 (42)	10.34 (21)	10.34 (21)	

*p* values indicate differences in the distribution of categorical variables between men and women (χ² test).

**Table 2.** Group size by industry and gender.

	All% (*n*)	Men% (*n*)	Women% (*n*)	*p* value
1 Managers	7.88 (16)	4.93 (10)	2.96 (6)	<.0001
2 Professionals	38.42 (78)	17.24 (35)	21.18 (43)	
3 Associate professionals	0.99 (2)	0	0.99 (2)	
4 Administrative and customer service workers	3.45 (7)	1.97 (4)	1.48 (3)	
5 Service, care, and sales workers	9.85 (20)	3.94 (8)	5.91 (12)	
6 Agricultural and forestry workers	7.39 (15)	3.94 (8)	3.45 (7)	
7 Building and manufacturing workers	5.42 (11)	3.94 (8)	1.48 (3)	
8 Mechanical manufacturing and transport workers	7.88 (16)	7.88 (16)	0	
9 Elementary occupations	18.72 (38)	11.33 (23)	7.39 (15)	

*p* values indicate differences in the distribution of categorical variables between men and women (χ² test).

In terms of education, the largest group was those with higher education (*n* = 89), while the smallest group had primary education (*n* = 6). Among women, the largest group was those with higher education (*n* = 53); similarly, among men, but this group was smaller (*n* = 36). Demographic characteristics of the participants, including gender, education status, and working on night shifts are shown in Table 1, with a distinction between job types.

In terms of industry, the largest group was professionals (*n* = 78), and the smallest group was associate professionals (*n* = 2). The number of women and men in each industry is shown in Table 2.

### The relationship between PA, working hours, smoking and physiological parameters

Spearman’s partial correlation analysis, controlling for gender, showed a significant positive relationship between self-reported weekly PA (IPAQ-SF-derived MET-min/week) and estimated *V*O_2max_. The partial correlation coefficient was *r*ₛ = 0.2339, with *p* = 0.0012.

In addition, an inverse relationship was observed between the number of hours worked per week and the FEV_1_/FVC level. The partial correlation coefficient was *r*ₛ = −0.1650, with *p* = 0.0269.

An inverse relationship was also observed between the number of cigarettes smoked daily and FEV_1._

The results are presented in Table 3.

**Table 3.** Partial spearman rank correlation coefficients between selected variables.

Occupational and lifestyle variables	Cardiorespiratory and pulmonary parameters	ρ (Spearman)	*p* value
MET-min/week	Estimated *V*O_2max_	0.2339	0.0012
working hours/week	Estimated *V*O_2max_	−0.05350	0.4647
Smoking	Estimated *V*O_2max_	−0.03942	0.5892
MET-min/week	FEV_1_	0.04417	0.5527
MET-min/week	FVC	0.09564	0.1978
MET-min/week	FEV_1_/FVC	−0.07406	0.3218
Working hours/week	FEV_1_	−0.08742	0.2380
Working hours/week	FVC	−0.02547	0.7314
Working hours/week	FEV_1_/FVC	−0.1650	0.0269
Smoking	FEV1	−0.16603	0.0239
Smoking	FVC	−0.10881	0.1404
Smoking	FEV_1_/FVC	−0.10792	0.1481

The gender variable was controlled.

Estimated *V*O_2max_ presented in mL/kg/min.

MET-min/week refers to self-reported weekly physical activity (IPAQ-SF-derived MET-min/week).

### Differences between blue-collar and white-collar workers and night shift and no-night shift workers

Blue-collar workers had significantly higher levels of self-reported weekly PA compared to white-collar workers (*p* = 0.0003), while estimated *V*O_2max_ values were significantly lower in the blue-collar group (*p* = 0.0467). Descriptive statistics for the general group (median and interquartile range) are presented in Table 4.

**Table 4.** Comparison of parameters across occupational groups and night-shift status; values are median (IQR).

	Jobtype				
Measure	White-collar (*n* = 125)		Blue-collar (*n* = 80)		
	Median	IQR	Median	IQR	*p* value
MET-min/week	1884	2403	3072	2625	0.0003
Estimated *V*O_2max_	24.31	8.36	23.37	8.65	0.0467
FEV_1_	94.00	17.50	92.5	19.00	0.4300
FVC	98.00	16.00	98.00	17.00	0.5997
FEV_1_/FVC	98.00	9.00	97.00	9.00	0.4011
Smoking	0.00	0.00	0.00	2.50	0.0229

Estimated *V*O_2max_ presented in mL/kg/min.

MET-min/week refers to self-reported weekly physical activity (IPAQ-SF-derived MET-min/week).

When stratified by sex, the difference in estimated *V*O_2max_ remained significant among women (blue-collar median, IQR = 23.26, 5.98; white-collar median, IQR = 26.27, 9.16; *p* = 0.0142) but not among men (blue-collar median, IQR = 23.42, 8.99; white-collar median, IQR= 22.58,7.59; *p* = 0.9144). These results suggest that the occupational difference in *V*O_2max_ is primarily driven by women.

In the case of self-reported weekly PA when stratified by sex, the difference remained significant among men (blue collar median, IQR = 3172.5, 2178.00; white collar median, IQR = 1866,2472.00; *p* = 0.0003) but not among women (blue collar median, IQR= 2746.5, 2600.4; white collar median, IQR = 2079, 2356.5 *p* = 0.2434).

No statistically significant differences were observed between the groups in terms of spirometric parameters, but blue-collar workers smoked more cigarettes daily. It is worth noting, however, that the vast majority of the overall group did not smoke at all (*n* = 163).

No differences were observed in the above parameters between individuals working night shifts and those without night shifts. The data are presented in Table 5.

**Table 5.** Comparison of parameters by night-shift work status; values are median (IQR).

	Night shifts				
Measure	No night shifts (*n* = 161)		Work with night shifts (*n* = 42)		
	Median	IQR	Median	IQR	*p* value
MET-min/week	2364	2528	3157.5	2826	0.2033
Estimated *V*O_2max_	23.80	7.95	24.69	9.33	0.5355
FEV_1_	94.00	17.00	93.00	22.00	0.8321
FVC	98.00	16.00	99.00	15.00	0.5585
FEV_1_/FVC	97.00	10.00	98.00	11.00	0.8502
Smoking	0.00	0.00	0.00	5.00	0.0694

Estimated *V*O_2max_ presented in mL/kg/min.

MET-min/week refers to self-reported weekly physical activity (IPAQ-SF-derived MET-min/week).

### Differences between industries and educational levels

The Kruskal–Wallis test revealed significant differences in medians in self-reported weekly PA between occupational groups (χ^2^(8) = 26.88, *p* = 0.0007).

Post-hoc analysis using the Dwass–Steel–Critchlow–Fligner method revealed that the *professionals* group differed significantly from the *agricultural and forestry workers* group (*p* = 0.0009) and the *elementary occupations* group (*p* = 0.0297). Representatives of the *professionals* group showed lower levels of self-reported weekly PA than the other two groups.

Descriptive statistics for industry groups are presented in Table 6.

**Table 6.** Comparison of parameters across industry groups; values are median (IQR).

	MET-min/week (*p* = 0.0007)	*V*O_2max_ *p* = (0.1074)	FEV1 *p* = (0.4066)	FVC *p* = (0.8470)	FEV1/FVC *p* = (0.7650)
1 Managers (*n* = 16)	1710.75 (2835)	22.26 (7.78)	104.00 (20.00)	102.00 (21.00)	98.00 (10.00)
2 Professionals (*n* = 78)	1866 (2088)	24.31 (7.41)	94.00 (17.00)	98.00 (16.00)	99.00 (10.00)
3 Associate professionals (*n* = 2)	1908 (1836)	30.76 (0.38)	86.50 (3.00)	95.00 (12.00)	91.50 (9.00)
4 Administrative and customer service workers (*n* = 7)	4266 (5202)	26.16 (14.10)	97.00 (6.00)	99.00 (8.00)	97.00 (13.00)
5 Service, care, and sales workers (*n* = 20)	2992.50 (3012)	23.16 (9.92)	89.00 (9.00)	93.00 (15.00)	97.00 (10.00)
6 Agricultural and forestry workers (*n* = 15)	4578 (3192)	23.84 (12.46)	94.00 (19.00)	98.00 (21.00)	94.50 (10.00)
7 Building and manufacturing workers (*n* = 11)	2559 (2948.4)	20.13 (9.77)	92.00 (33.00)	99.50 (25.00)	95.50 (13.00)
8 Mechanical manufacturing and transport workers (*n* = 16)	2346 (2799)	26.05 (6.69)	97.50 (15.00)	96.00 (17.50)	100.00 (8.00)
9 Elementary occupations (*n* = 38)	3172.5 (2079)	21.99 (5.88)	91.00 (18.00)	96.00 (18.00)	95.00 (10.00)

Estimated *V*O_2max_ presented in mL/kg/min.

MET-min/week refers to self-reported weekly physical activity (IPAQ-SF-derived MET-min/week).

No significant differences were found between the other groups.

When stratified by sex, as with blue-collar and white-collar workers, these results were significant for men (*p* = 0.0007) but not for women (*p* = 0.4165). The male *professionals* group was characterized by a significantly lower self-reported weekly PA (median = 1413; IQR = 1977) than the *elementary occupations* group (median = 3306, IQR = 1500) and *agricultural and forestry* workers (median = 5811; IQR = 3258).

The Kruskal–Wallis test revealed significant differences in self-reported weekly PA between education groups (χ^2^(4) = 11.48; *p* = 0.0217).

In the post hoc analysis using the Dwass–Steel–Critchlow–Fligner method, no significant differences were found between pairs of groups (all *p* > 0.05), although a trend towards higher MET-min/week values was observed in people with higher education (*p* ≈ 0.09).

Descriptive statistics for education groups are presented in Table 7.

**Table 7.** Comparison of parameters across education groups; values are median (IQR).

	MET-min/week (*p* = 0.0217)	*V*O_2max_ *p* = (0.1649)	FEV1 *p* = (0.7102)	FVC *p* = (0.4795)	FEV1/FVC *p* = (0.7439)
Primary education (*n* = 6)	3652.50 (2682.00)	21.51 (13.24)	88.50 (16.00)	97.00 (6.00)	95.50 (14.00)
Basic vocational education (*n* = 34)	2965.50 (2562.00)	21.48 (6.21)	91.50 (18.00)	93.50 (18.00)	98.50 (10.00)
Secondary trade education (*n* = 25)	2853.00 (2292.00)	23.26 (9.83)	92.00 (16.00)	99.00 (15.00)	97.00 (13.00)
Secondary education (*n* = 49)	2919.00 (3160.20)	23.84 (9.37)	92.50 (19.00)	98.50 (19.00)	97.00 (10.00)
Higher education (*n* = 89)	1664.50 (2457.75)	24.76 (7.55)	94.00 (17.00)	98.00 (14.00)	98.00 (9.00)

Estimated *V*O_2max_ presented in mL/kg/min.

MET-min/week refers to self-reported weekly physical activity (IPAQ-SF-derived MET-min/week).

When performing separate analyses for women and men, it was observed that these differences were significant for men (*p* = 0.0389), and in the post hoc analysis, significant differences were found between the secondary trade education group (median = 2853; IQR = 2478.00) and the higher education group (median = 1348; IQR = 2346.00; *p* = 0.0393). No significant differences were found in women.

## Discussion

To our knowledge, this is the first population-based study of Polish workers that examines CRF, as measured by estimated *V*O_2max_, across nine distinct occupational categories. In addition, it simultaneously checks CRF and spirometry, which is rare in occupational health research. Due to strict inclusion and exclusion criteria, the study cohort was relatively homogeneous in terms of health status. Consequently, the observed differences in CRF and pulmonary function are unlikely to be substantially influenced by major confounders such as comorbidities or smoking.

The study suggests associations between job type, industry, and education in the context of self-reported weekly PA, and between job type and estimated *V*O_2max_, with slight gender-specific differences observed in these results.

Blue-collar workers reported higher self-reported weekly PA, and this result was significant for men, but not for women. In contrast, no significant differences were observed in estimated *V*O_2max_ in men, while female blue-collar workers had lower values than white-collar workers. Given that *V*O_2max_ was estimated using the submaximal Astrand–Rhyming test, small observed differences, particularly between sexes, should be interpreted cautiously as they may fall within the measurement error of the test.

Also in men, significantly higher self-reported weekly PA was observed in the *agricultural and forestry workers* group (the highest) and the *elementary occupations* group than in the *professionals* group, which showed the lowest activity among all groups. These results are consistent with the results between white-collar and blue-collar workers. No significant differences were observed in women. These findings underscore the importance of objective physiological assessment, as self-reported PA does not fully predict CRF in occupational groups.

The reasons for high self-reported weekly PA and no differences in estimated *V*O_2max_ in male blue-collar workers may include, among others, overestimation of work intensity in the PA survey – the work may not be physically demanding enough to have a beneficial effect on *V*O_2max_ levels. This is confirmed by Korshøj’s study of a group of cleaners, which showed that despite high PA at work, its intensity was not sufficient to improve CRF. Such work is often characterized by relatively high occupational cardio-respiratory workload and minimal high-intensity cardiorespiratory activity [39].

Epidemiological studies show that high levels of PA at work increase the risk of cardiovascular disease and mortality, even after taking into account other risk factors, including socio-economic status, leisure-time exercise, and other health-promoting behaviours [40]. These findings can be discussed in the context of the PA paradox, a concept suggesting that there are opposing health effects of occupational PA and leisure-time PA – occupational PA and leisure-time PA have different health outcomes with leisure-time PA generally being linked to more favourable health profiles [18,40].

Among women, blue-collar workers did not show higher self-reported weekly PA, but lower estimated *V*O_2max_ than white-collar women workers.

These gender differences may partly reflect differences in the type and intensity of occupational tasks performed by men and women, as men are more likely than women to perform physically demanding work, causing different stress on the cardiovascular system [41].

Physical work performed by women is often characterized by a different type of activity - it involves prolonged standing, repetitive movements, and manual labour. These activities may not sufficiently stimulate the circulatory and respiratory systems to induce measurable physiological adaptations. This observation supports the hypothesis that lifestyle factors and PA performed outside of work may play a particularly important role in cardiorespiratory fitness. PA at work, prolonged static postures in particular that are performed over long periods of time (often >40 h/week), with insufficient time for recovery, have been suggested to raise heart rate and blood pressure. On the other hand, leisure time PA typically takes place in short moderate or high intensity bouts of predominantly aerobic activities, accompanied by much longer recovery periods, which may influence beneficial physiological changes [41].

Given the importance of CRF as a predictor of morbidity and mortality, the lower estimated *V*O_2max_ in female blue-collar workers and the lack of differences in self-reported PA may indicate the role of other factors, including those related to lifestyle. A systematic review by Elser et al. suggests a similar but more general trend – generally poorer health was observed in women who were blue-collar workers compared to men in blue-collar occupations and other women. However, the authors emphasize substantial differences between populations in the studies included in the review – geographical differences may influence labour regimes, gendered norms, and thus the results of the studies [23]. Previous studies have also reported that women in physically demanding occupations more frequently report exposure to workplace stressors and stress-related symptoms, such as fatigue and psychosomatic complaints [42,43]. Stress has a biological impact on health through influencing the autonomic nervous system and cortisol dynamics, hypothalamic–pituitary–adrenal axis regulation, and gene expression [44]. Furthermore, the study by St-Pierre et al. indicated a link between stress and unhealthy behaviours – PA and greater screen sedentary behavior [45].

Considering this issue, regardless of gender, blue-collar workers’ health may be at a generally higher risk than that of white-collar employees, potentially reflecting greater exposure to heavy physical work, noise, and harmful chemicals. In addition, previous studies have suggested that they are usually less engaged in activities that promote health, which could contribute to their overall vulnerability [46].

In terms of educational attainment, significant results were observed among men – the lowest self-reported PA was observed in the group with higher education, while the highest was in the primary education group. This pattern may reflect differences in occupational characteristics– most people with higher education are white-collar workers, while the majority of those with higher self-reported PA are blue-collar workers. Furthermore, the small size of some groups, particularly those with basic education, suggests caution in concluding.

Despite the limited availability of data on the relationship between education level and CRF, it is evident that these values may vary depending on the country in which the population is studied. For example, Vaisanen et al. reported that low-skilled workers had lower CRF compared to high-skilled workers, with the differences between white- and blue-collar workers being less pronounced [18].

It was observed that blue-collar workers reported smoking more cigarettes per day than white-collar workers. However, the number of smokers in the overall group was very small. Among blue-collar workers, 22 people smoked, and among white-collar workers [19]. This could partly explain the fact that no significant associations were observed between blue-collar and white-collar workers regarding spirometry results. The results of spirometry tests were significant in terms of the number of hours worked per week and FEV_1_/FVC%, but the effect was very small, so working hours alone explain very little of the variability in FEV_1_/FVC% values. In the study of other relationships between job type, education, number of hours worked, and spirometry parameters, no statistically significant differences were found. Similarly, in the study by Trzmiel et al. conducted on retired workers, no significant differences were observed in most spirometry parameters, with the exception of FEV1 [14]. The lack of differences may partly reflect increasingly comprehensive regulations on working conditions regarding general protective measures, reduction and elimination of exposures, personal respiratory protection, as well as environmental and medical surveillance. Has been suggested by the authors of studies on the Norwegian population, which may be relevant to Poland in recent years [47].

## Strengths and limitations

This study presents several important strengths that enhance the reliability and applicability of its findings. First, it assesses CRF—a well-established predictor of morbidity and mortality—using a method that is both cost-effective and easily accessible. This enhances the potential for future implementation in various occupational health settings. The number of scientific studies on the impact of working conditions on health parameters in the Polish population of working adults is minimal. There are also a few publications in English-language databases describing physical performance depending on working conditions.

Second, the study sample includes individuals employed across a wide range of industries, educational backgrounds, and socioeconomic statuses. The randomness of employment status adds to the diversity of the sample, increasing the representativeness and generalizability of the findings within the national context.

Finally, procedures such as participant qualification, questionnaire administration, and CRF testing were carried out by a single trained individual. This consistency helps reduce inter-rater variability and increases the internal validity of the data collected.

This study has some limitations. The first limitation of this study relates to the generalizability of its findings. While the results reflect the experiences of Polish workers, they may not apply to individuals from other countries. Additionally, differences in labor culture across nations may hinder the equitable application of these findings in an international context.

Limitations include its cross-sectional design, which prohibits causal inferences to future disease incidence. Furthermore, a limitation of this study is that it relies on self-reported PA assessed using the IPAQ-SF questionnaire. This tool may overestimate PA levels, particularly for occupations involving physical exertion, and does not distinguish between PA performed at work and PA performed during leisure time. Therefore, this self-reported PA questionnaire may not accurately reflect the cardiorespiratory stimulus resulting from the activity, and the observed discrepancy between self-reported PA and estimated *V*O_2max_ may be partly due to measurement limitations.

Finally, despite detailed inclusion criteria aimed at selecting subjects at a similar functional level, postoperative condition, although well-functioning, ready to come back to work, may constitute a bias in the study.

In addition, for some groups, the number of people was small, particularly in the *Associate Professionals* occupational group and the primary education group. Such differences may be related to the increasing development of the country and the improving level of education – nowadays, few people in Poland under the age of 70 have only primary education.

## Conclusions

The study observed differences in self-reported weekly PA and estimated *V*O_2max_ across occupational groups and gender. Among men, blue-collar workers reported higher self-reported PA than white-collar workers, with no corresponding differences in estimated *V*O_2max_. Among women, blue-collar workers did not report higher PA but had lower estimated *V*O_2max_ than white-collar women; however, these differences were small and should be interpreted cautiously due to the limitations of submaximal estimation.

No consistent associations were observed between occupational characteristics and spirometry parameters, with only a very weak relationship between working hours and FEV_1_/FVC%, suggesting limited occupational influence on pulmonary function in this cohort.

Overall, the findings suggest that occupational PA alone may not be sufficient to induce measurable improvements in CRF. It also highlights the value of jointly assessing CRF and pulmonary function when examining occupational health. Given the cross-sectional design and small subgroup sizes in some cases, further research is needed to confirm these observations, particularly among women in blue-collar occupations.

## Data Availability

The data presented in this study are available on request from the corresponding author. The data are not publicly available due to not obtaining consent from respondents to publish the data.
